# Supplementary material for: Antibiotic susceptibility and resistance genes profiles of Vagococcus salmoninarum in a rainbow trout (Oncorhyncus mykiss, Walbaum) farm
Source: PeerJ. 2024 Mar 28;12:e17194. doi: 10.7717/peerj.17194 (PMC10981890; doi:10.7717/peerj.17194)
Supplement: Supplemental Information 1 [file peerj-12-17194-s001.docx]

**Table S1.** Biochemical characterization of *Vagococcus salmoninarum* isolates

| **Characteristics** | **Isolates** | **Reference** | **Characteristics** | **Isolates** | **Reference** |
| --- | --- | --- | --- | --- | --- |
| Colony size | <1 mm | <1 mm | D-Glucose | + | + |
| Gram staining | + | + | D-Fructose | + | + |
| Shape | cb | cb | D-Mannose | + | + |
| Motility | - | - | L-Sorbose | - | - |
| Hemolysis | α | α | Rhamnose | - | - |
| Oxidase | - | - | Dulcitol | - | - |
| Catalase | - | - | Inositol | - | - |
| O/F | F | F | α-Methylxy-D-mannoside | - | - |
| Urease | - | - | α-Methylxy-D-glucoside | - | - |
| Indole | - | - | N-Acetylglucosamine | + | + |
| NO3 reduction | - | - | Amygdalin | + | + |
| Aesculin hydrolysis | + | + | Arbutin | + | + |
| H2S production on TSI | - | - | Salicin | + | + |
| Hippurate hydrolysis | - | - | Cellobiose | + | + |
| Pyrrolidonyl arylamidase | + | + | Maltose | - | - |
| α-Galactosidase | - | - | Melibiose | - | - |
| β-Glucuronidase | - | - | Saccharose | + | + |
| β-Galactosidase | - | - | Melezitose | - | - |
| Alkaline phosphatase | - | - | Xylitol | - | - |
| Leucine arylamidase | - |  | β-Gentiobiose | + | - |
| Arginine dihydrolase | - | - | D-Turanose | - | - |
| Ribose | + | + | D-Lyxose | - | - |
| **Carbon source utilization assays**  Mannitol | - | - | D-Tagatose | + | + |
| Sorbitol | - | - | D-Fucose | - | - |
| Lactose | - | - | L-Fucose | - | - |
| Trehalose | + | + | D-Arabitol | - | - |
| Inulin | - | - | L-Arabitol | - | - |
| Raffinose | - | - | Potassium gluconate | - | - |
| Starch | - | - | Potassium 2-ketogluconate | - | - |
| Glycogen | - | - | Potassium 5-ketogluconate | - | - |
| Glycerol | - | - | **Growth at:** |  |  |
| Erythritol | - | - | at 10°C | + | + |
| D-Arabinose | - | - | at 20°C | + | + |
| L-Arabinose | - | - | at 37°C | + | + |
| D-Xylose | - | - | at 42°C | - | - |
| L-Xylose | - | - | **Chemical sensitivity assays**  at pH 9.6 | + | + |
| Adonitol | - | - | in 6.5% NaCl | - | - |
| β-Methylxylidose | - | - | **Growth on** |  |  |
| Galactose | - | - | MacConkey Agar | - | - |

-, negative; +, positive; cb, coccobacilli; O/F, Oxidation-Fermentation; F: Fermentative; α, Alpha hemolysis; v, variable character; NA, not evaluated.
